# Grafting of bacterial cellulose nanofibers with polylactic acid (PLA) enables homogeneous dispersion in PLA and improved strength and stiffness without loss of ductility

**DOI:** 10.1039/d6ra04355k

**Published:** 2026-07-10

**Authors:** Yuuki Takatsuna, Erik Reimhult, Ronald Zirbs

**Affiliations:** a Institute of Colloid and Biointerface Science, BOKU University Vienna Austria ronald.zirbs@boku.ac.at erik.reimhult@boku.ac.at

## Abstract

The development of sustainable polymer materials is a major challenge, driven by the increasing demand for environmentally sustainable alternatives to conventional plastics. Poly(lactic acid) (PLA), derived from renewable resources and exhibiting biodegradability, has emerged as a promising candidate. Although PLA exhibits favorable properties, its mechanical performance remains insufficient for many applications. A common strategy to improve performance is to incorporate stiff nanofillers, such as nanocellulose, to compensate for the limitations of neat PLA. However, PLA typically exhibits a trade-off between strength and ductility, and the addition of stiff fillers often further reduces ductility. Here, we show that interfacial design *via* PLA grafting onto bacterial cellulose enables nanoscale dispersion of cellulose nanofillers, providing an effective strategy for mitigating the strength–ductility trade-off in PLA composites. This approach resulted in simultaneous improvements in strength and Young's modulus by 44% and 52%, respectively, without compromising ductility. 3D analysis using serial block-face scanning electron microscopy enabled direct visualization and quantitative characterization of the dispersion structure and failure modes, suggesting that the improvements result from controlled cavitation on the nanofibers and obstruction of void growth due to improved nanofiber-matrix adhesion. These findings highlight the critical role of nanoscale dispersion and interfacial design in influencing the mechanical performance of polymer nanocomposites. This strategy provides a framework for designing high-performance biodegradable materials with balanced mechanical properties.

## Abstract 2

The development of sustainable polymer materials is a major challenge, driven by the increasing demand for environmentally sustainable alternatives to conventional plastics.

## Graphical abstract

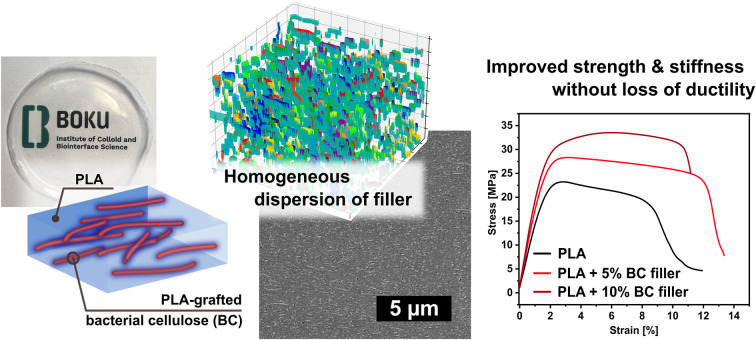

## Introduction

Increasing plastic waste and the continued dependence on fossil-resource-based polymers have raised serious environmental concerns.^1^ In response, there is a growing demand for sustainable materials derived from renewable resources, particularly those that are biodegradable.^2,3^ Polylactic acid (PLA) has emerged as one of the most promising candidates due to its biodegradability, transparency, and industrial processability. As a result, PLA is increasingly used in applications such as packaging, molded products, and biomedical applications.^4–6^

For many of these applications, however, PLA materials must exhibit sufficient mechanical performance, particularly strength and stiffness. Reinforcement through the addition of fillers is a widely studied strategy to enhance these properties.^7^ Yet, despite its effectiveness, this approach frequently encounters a major challenge: improvements in strength or stiffness often reduce ductility.^6,8^ This strength/stiffness–ductility trade-off significantly limits the practical implementation of PLA composites. The inherently brittle nature of PLA further exacerbates this issue, making the loss of ductility particularly detrimental.

The strength/stiffness–ductility trade-off in PLA composites is influenced by multiple factors, and interfacial characteristics and dispersion state are considered among the important contributors.^9–11^ Among these factors, this study places particular emphasis on understanding the roles of interfacial interactions and nanoscale dispersion. Incompatibility between hydrophilic fillers and hydrophobic PLA causes aggregation or interfacial debonding,^12–14^ both of which generate stress concentrations that promote premature failure. These problems become especially pronounced for nanoscale fillers, where the total high-surface-energy area is large, and dispersion is difficult to maintain.^15^ Therefore, the design of interfacial interactions and the control of nanoscale dispersion are essential for mitigating the strength–ductility trade-off in PLA nanocomposites.

We selected bacterial cellulose (BC) as a model nanofiller; it is a highly pure, mechanically robust, and biodegradable nanofiber produced through microbial fermentation.^16^ In contrast, plant-derived cellulose materials may contain residual lignin and hemicellulose. Hence, composites based on plant-derived cellulose with variable physical and chemical properties make it difficult to isolate the influence of surface modification of cellulose nanofibers on particle dispersion and composite mechanical properties. Owing to its high stiffness and strength, BC is considered a promising reinforcing filler for PLA. However, the cellulose surface is highly hydrophilic and exhibits poor affinity with PLA. Various surface modification strategies for cellulose nanofillers have been proposed to improve compatibility with PLA.^7^ Although hydrophobization treatments such as acetylation or silanization can improve the dispersibility of cellulose nanofillers, studies report that increases in tensile strength and Young's modulus are achieved at the expense of elongation at break.^9,17–20^ This trend indicates that the interfacial strength remains insufficient to maintain ductility. The limited interfacial strength is particularly critical when highly rigid fillers such as cellulose nanofibers are incorporated. As an alternative approach, grafting PLA or oligomeric lactic acid (OLA) chains onto nanocellulose has been explored, leveraging the chemical affinity between PLA and the grafted chains.^7,21^ OLA grafting does not completely shield the interactions between nanocellulose cores and does not provide the same interfacial interactions as between PLA chains. Hence, aggregation is not expected to be completely suppressed, which will be more pronounced for nanofibers than for nanocellulose particles. Such heterogeneous dispersion can lead to localized stress concentration and may contribute to the commonly observed reduction in ductility in nanocellulose-reinforced PLA systems.^22,23^

To remedy this, we grafted PLA chains onto the surface of BC fibers to improve interfacial compatibility and promote homogeneous nanoscale dispersion. High-aspect-ratio nanofillers are expected to provide more efficient mechanical reinforcement than their low-aspect-ratio counterparts, as demonstrated in previous studies.^24,25^ Therefore, the present interfacial design aims to enable efficient stress transfer for effective mechanical reinforcement while mitigating the ductility loss typically observed in nanocellulose–PLA composites, making PLA grafting onto high-aspect-ratio BC a logical materials design strategy for this purpose.

To directly verify how interfacial design affects dispersion and, in turn, mechanical properties, we performed serial block-face scanning electron microscopy (SBF-SEM) before and after fracture. SBF-SEM allows direct nanoscale visualization of the three-dimensional distribution of BC within the PLA matrix. It provides a unique opportunity to link interfacial properties to dispersion without model assumptions. Furthermore, it provides critical insights into the deformation and fracture mechanisms in the resulting nanocomposites, especially in systems where nanoscale dispersion and morphology, for which most other methods lack the resolution, are expected to contribute to mechanical performance.

Our objective was to evaluate the effectiveness of PLA-grafted BC nanofibers as a reinforcing filler for PLA, with a focus on (i) achieving a favorable balance between strength, stiffness, and ductility, (ii) investigating the role of interfacial interactions, and (iii) correlating nanoscale dispersion and fracture behavior through SBF-SEM analysis.

## Experimental

### Materials

PLLA was purchased from Biomer (L9000, *M*_w_ ≥ 150 kg mol^−1^, D-content ≈ 1.5%). Lactic acid (Aldrich, solute concentration: 85 wt% aqueous solution), tin(ii) 2-ethylhexanoate (Sn(Oct)_2_, Aldrich, ≥85% purity), dichloromethane, chloroform, and alcohol were purchased from Sigma-Aldrich. The raw bacterial cellulose material was SCOBY, a by-product of kombucha fermentation.

### Methods

#### BC preparation

100 mL of fully fermented kombucha tea (sucrose content <5%) and approximately 10 vol% of previously produced SCOBY were mixed with 10 g L^−1^ of agricultural waste obtained after boiling leaves and mixed biowaste (organic household waste) for 10 min, and adding 100 g L^−1^ of sucrose. The fermentation was carried out for 15 days at room temperature, resulting in SCOBY production containing bacterial cellulose. 1000 g of SCOBY was blended for 3 min using a blender (Nutri-Blender MAX Hochleistungsmixer 2000W, Munich, Germany) and then centrifuged. The blended BC was redispersed in deionized water and centrifuged again to remove water-soluble impurities. The remaining microorganisms and soluble polysaccharides were removed by stirring in a 0.1 M NaOH aqueous solution at 80 °C for 20 min. The purified BC was then centrifuged three times and washed to neutral pH. The purified BC was stored as a water dispersion at approximately 3% concentration.

#### Grafting of lactic acid chains onto BC

250 g of purified bacterial cellulose dispersion in water were mixed with 880 g of lactic acid. The mixture was mechanically stirred and heated at 100 °C under 850 mbar to remove most of the water content. Subsequently, the pressure was reduced to 20 mbar, and the mixture was stirred at 120 °C for 2 h. While heating from 120 °C to 160 °C at a rate of 5 K h^−1^, 5.6 mL of Sn(Oct)_2_ were added at 130 °C as a catalyst. After reaching 160 °C, the mixture was further stirred to complete a total reaction time of 4 h. After cooling to room temperature, the crude product was dissolved in dichloromethane (DCM), and PLA-grafted BC was separated from free PLA by centrifugation. The purification procedure, including DCM washing and centrifugation, was repeated three times. The crude PLA-modified BC was redispersed in chloroform. The dispersion was then washed with 1 M HCl in a separatory funnel, followed by washing with deionized water until the solution reached neutral pH. The organic layer was dried by rotary evaporation under reduced pressure, yielding the final product denoted as BC-*g*-PLA.

#### Preparation of PLA film

Films of neat PLA and PLA/BC composite materials were fabricated using solvent casting. For all samples, the solution was prepared to achieve a total solid content of 10 wt% (PLA + filler) in chloroform, which was selected because its lower volatility compared to DCM allowed more controlled solvent evaporation and more homogeneous film formation. PLA pellets were then added to the dispersion and stirred overnight. The resulting solution was poured into a round silicon mold. After applying a 400 mbar vacuum to remove bubbles, the solvent was evaporated in a fume hood to obtain BC/PLA composite films.

For the unmodified BC, the aqueous BC dispersion was first subjected to solvent exchange using ethanol, isopropanol, and dichloromethane, each performed three times, before mixing with PLA in chloroform for solvent casting. The sample names used in this study consist of the filler type followed by its loading (wt%), for example, PLA/BC-*g*-PLA5 or PLA/BC10.

### Characterizations

#### Fourier transform infrared spectroscopy (FT-IR)

The molecular structures of the filler materials were confirmed by FT-IR using an FT-IR ATR spectrometer (Vertex 70, Bruker Austria GmbH, Vienna, Austria), with 32 scans in the wavelength range of 400–4000 cm^−1^ in transmission mode. Substances were mounted directly onto the ATR unit and measured using the pressure stamp.

#### Thermogravimetric analysis (TGA)

TGA was performed on a Mettler Toledo TGA/DSC (Mettler Toledo GmbH, Vienna, Austria) under a nitrogen atmosphere (80 mL min^−1^ nitrogen). The measurement was carried out over the temperature range 25–650 °C at a heating rate of 10 K min^−1^. The resulting DTG diagrams were analyzed using Origin (OriginLab, MA, USA) with the peak fitting function to calculate the fractions of cellulose and PLA. The maximum degradation temperature (*T*_max_) was defined as the temperature corresponding to the peak of the DTG curve.

#### Differential scanning calorimetry (DSC)

The non-isothermal behavior of PLA composite films was evaluated by DSC on a Mettler Toledo TGA/DSC (Mettler Toledo GmbH, Vienna, Austria) under a nitrogen atmosphere. Samples of 8.5 ± 0.5 mg were heated from 25 to 190 °C and held isothermally at 190 °C for 3 min to eliminate the thermal history. The samples were cooled to 25 °C and reheated to 190 °C. The cooling and heating rate was 10 K min^−1^. The first and second heating curves were recorded.

The crystallinity of the PLA phases was calculated by:*χ*_c_[%] = (Δ*H*_m_ − Δ*H*_cc_/Δ*H*^0^_m_ × *w*_f_) × 100[%]where Δ*H*_m_ is the melting enthalpy, Δ*H*_cc_ is the cold crystallization enthalpy, *w*_f_ is the weight fraction of polymer and Δ*H*^0^_m_ is the melting enthalpy of completely crystallized PLA (93 J g^−1^).

#### Nuclear magnetic resonance (NMR)

^1^H NMR spectra were recorded on a Bruker AV III 300 MHz spectrometer (Bruker Company, Switzerland) in dimethyl sulfoxide-d_6_ (DMSO-d_6_) as solvent. The samples were measured at 25 °C at a solute concentration of 10 mg mL^−1^. The sample was dispersed in DMSO-d_6_ and sonicated for 60 min prior to measurement.

#### Serial block-face SEM (SBF-SEM)

The fragments of nanocomposite films were trimmed with a glass knife on a UC-7 ultramicrotome (Leica Microsystems, Vienna, Austria) to 500 µm × 500 µm × 500 µm and mounted on the microtome of the Apreo SEM (Thermo Fisher Scientific, Netherlands). Scans were acquired at 1 kV and 50 pA. The *in situ* microtome was used to remove layers of 150 nm thickness, and each exposed surface was imaged. The resulting image stacks were processed using the ImageJ software (NIH, Bethesda, MD, USA). Filler volume fraction measurements and 3D reconstruction were performed using Amira (Thermo Fisher Scientific, Waltham, MA, USA).

#### Mechanical tests

Tensile tests were performed on a universal dual-column testing frame (Series 5969, Instron Ltd, Norwood, UK). The specimens were prepared by cutting the films (thickness: 180 µm) into ISO 527-2 Type 1BA geometry using a laser cutter. This specimen type was selected due to the limited film size available for composite preparation. At least five specimens in each group were tested. The strain was recorded using a video extensometer (IMT-CAMO018, camera: GigE PoE, IMETRUM, UK) tracking black dots painted on “dog-bone” specimens with an opaque black permanent marker. The testing speed was 50 mm min^−1^.

#### Image analysis

The SBF-SEM and cross-section image analyses were performed using Amira software to encode whether a voxel contained a particle, and Python scripts to stitch occupied voxels into particles, label particles, and analyze their properties and spatial organization. Individual particles were stitched together and uniquely labeled from the binary voxel data using a 3D connected-component labeling algorithm, without a morphological closing step. Volume fractions were calculated based on the particle voxel occupancy data. The orientation of each particle was calculated by selecting the eigenvector of the covariance matrix of the mass distribution of the particle with the largest eigenvalue. These orientational vectors were used to calculate the nematic order parameter locally for each particle with its 15 nearest neighbors, as well as globally. Particles comprising fewer than 10 voxels were discarded from these calculations as lacking a meaningful orientation. A more extensive description of the logic, algorithms, and parameters used can be found in the SI, along with additional parameters analyzed.

Void-particle co-localization was evaluated by comparing the observed mean void-to-particle distance with a physically constrained random baseline across batches of cross-sectional images after mechanical testing. Details of the image analysis pipeline and tables of fitting parameters are described in the SI. *Z*-Scores (standard deviations away from an expected random spatial association) and one-sided empirical *p*-values (the fraction of simulations closer than observed) were calculated for the difference between the measured distance and the random statistical distribution to quantify whether voids preferentially interact with particles. Morphological, nematic order, and co-ordering parameters were also calculated from the binary masks of voids and particles. Calculations were performed for all detected voids and only voids larger than 50 pixels, respectively. Mean *Z*-scores and *p*-values were calculated from batches of cross-section images with Stouffer's formula for >22 000 particles and >12 000 voids for PLA/BC-*g*-PLA5 and >235 000 particles and >76 000 voids for PLA/BC-*g*-PLA10. See SI for further information on logic, algorithms, fitting parameters, and additional calculated parameters.

#### Water vapor transmission rate (WVTR) measurements

WVTR was evaluated using a custom-built measurement system. A beaker containing 40 mL of deionized water was sealed with a 3 cm-diameter film using a hot glue gun. This assembly was then placed in a container pre-filled with desiccated silica gel and incubated at 37 °C for 24 h. The WVTR was determined by measuring the weight difference of the sealed beaker before and after incubation.

#### Biodegradability tests

Biodegradability tests were conducted on composite films approximately 60 µm thick. The films were buried in compost placed in a 10 cm × 10 cm glass container and maintained at 60 °C. During the first three weeks, the films could be removed without damage and were rinsed with water to examine surface changes. After this period, the films became too brittle to be removed intact; thus, the compost was carefully removed to observe the remaining film fragments. The surface morphologies of sample pieces were analyzed by SEM after three weeks.

## Results and discussion

### Surface modification of BC *via* PLA grafting

**Fig. 1:**
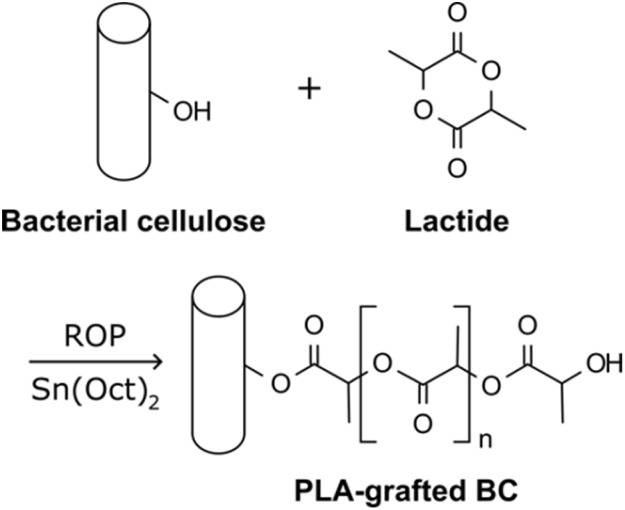
Schematic illustration of PLA grafting onto bacterial cellulose (BC) through Sn(Oct)_2_-catalyzed ring-opening polymerization of lactide from hydroxyl groups on the BC surface.

To enhance compatibility with the PLA matrix and to promote uniform nanoscale dispersion, we grafted PLA chains onto bacterial cellulose (Fig. 1). This modification was achieved by dispersing BC in lactic acid and removing water by heating, during which lactide was formed *in situ*. Ring-opening polymerization of the lactide was then initiated from hydroxyl groups on the BC surface using Sn(Oct)_2_ as a catalyst. After the grafting reaction, ungrafted PLA chains were removed by repeated dispersion in DCM followed by centrifugation, yielding purified PLA-grafted BC (BC-*g*-PLA), which was characterized and used to prepare composite films.

**Fig. 2:**
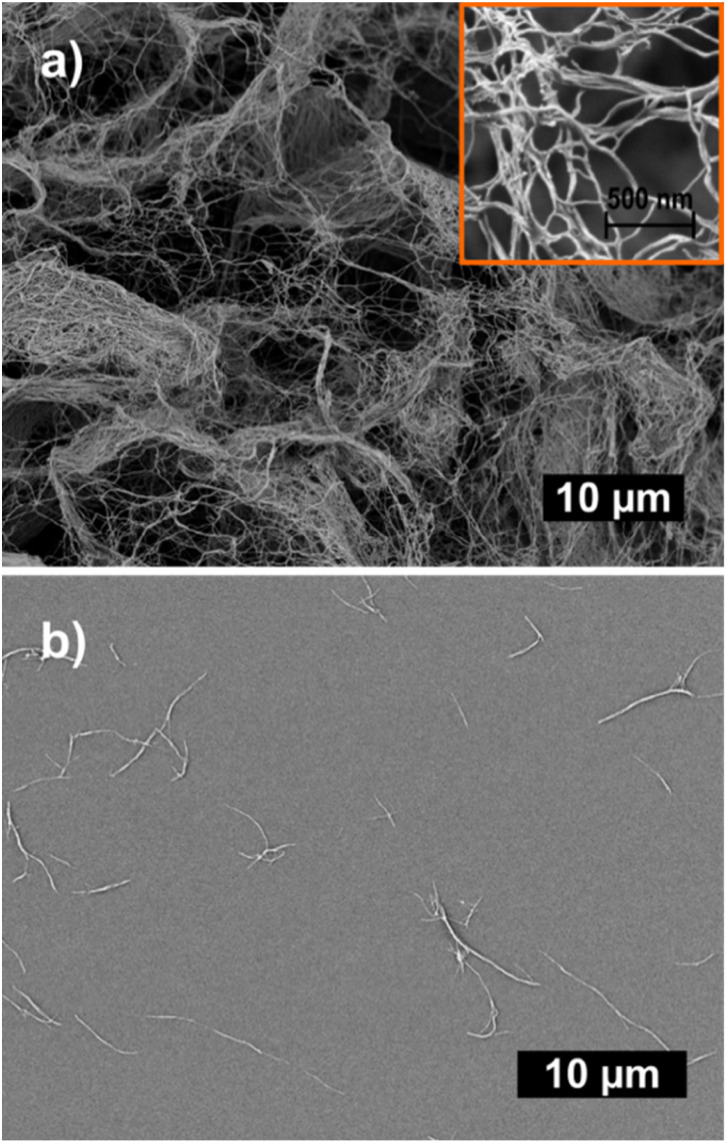
SEM images of (a) purified bacterial cellulose after freeze-drying from a *tert*-butanol suspension, and (b) BC-*g*-PLA obtained from a diluted dispersion in DCM spread on a silicon wafer.

The scanning electron microscopy (SEM) images shown in Fig. 2 compare BC before and after surface modification. The unmodified BC was freeze-dried after solvent exchange of its aqueous dispersion with tBuOH. In contrast, the surface-modified BC was redispersed in DCM and then dried on a silicon wafer. BC fibers with diameters of 38 ± 12 nm and lengths of approximately 3.1 ± 1.7 µm were determined based on the analysis of 300 individual fibers from the TEM images. These results demonstrate that the surface-modified BC maintains its nanofiber structure and that PLA has been successfully grafted onto the fibers.

**Fig. 3:**
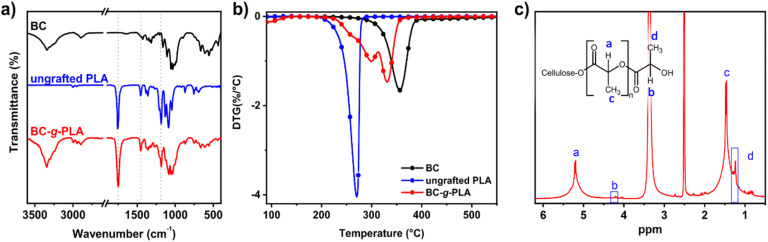
Characterization of BC-*g*-PLA: (a) FTIR spectra of unmodified BC, BC-*g*-PLA and ungrafted PLA obtained from the same grafting reaction, showing characteristic peaks associated with PLA, (b) DTG curves demonstrating the thermal degradation behavior, and (c) ^1^H NMR spectra confirming the presence of PLA signals.

The purified BC-*g*-PLA was analyzed by FTIR to confirm its chemical composition. As shown in Fig. 3a, the surface-modified BC filler exhibited peaks around 1750, 1450, and 1200 cm^−1^, which are characteristic of PLA. These correspond to the C=O stretching mode, the methyl asymmetric deformation mode, and the symmetric C–O–C stretching modes of the ester, respectively. Peaks attributed to PLA were also observed in the NMR analysis (Fig. 3c), confirming successful grafting.

As shown in Fig. 3b, the TGA of the surface-modified BC showed a two-step thermal decomposition, which was attributed to the decomposition of the grafted PLA and the decomposition of the BC. Approximately 57% of the weight of the surface-modified BC was composed of grafted PLA according to the peak area ratio in the DTG curve. This corresponds to a grafted PLA molecular weight of approximately 8–15 kDa, which is consistent with the number-average molecular weight (*M*_*n*_) of 15 kDa for the free PLA obtained from the same synthesis. The molecular weight range of the grafted PLA chains was calculated from the specific surface area of the BC nanofibers, using the fiber diameter range determined by TEM and assuming a uniform cylindrical morphology. A grafting density of 1 chain per nm^2^ was assumed as an approximate surface hydroxyl density, since the size of a glucose unit in cellulose is approximately 1 nm^2^, and the primary hydroxyl group at the C6 position is generally considered to be the most reactive due to its greater accessibility and lower steric hindrance compared to the secondary hydroxyls at the C2 and C3 positions.^26^

### Structure and properties of PLA composites with BC-based fillers

To investigate the effect of BC-based fillers, composite films containing 0, 1, 5, and 10 wt% of either unmodified BC or BC-*g*-PLA were prepared by solvent casting (Table 1). These films were evaluated by SEM, TGA, DSC, and tensile, biodegradation, and gas-barrier tests. Unmodified BC was included as a control to evaluate the effect of PLA grafting on filler dispersion.

**Table 1:** Composition and designation of PLA and PLA composite films, including filler type, loading, maximum degradation temperature (*T*_max_), and crystallinity (*χ*_c_)^a^

Sample	Filler	Loading [wt%]	*T*_max_ [°C]	*χ*_c_ [%]	
				1 cycle	2 cycle
PLA	—	—	367.1	14.4	0.1
PLA/BC1	Unmodified BC	1	369.5	16.5	30.9
PLA/BC5	Unmodified BC	5	371.5	26.7	28.4
PLA/BC10	Unmodified BC	10	368.3	20.9	25.9
PLA/BC-*g*-PLA1	BC-*g*-PLA	1	368.8	15.3	7.6
PLA/BC-*g*-PLA5	BC-*g*-PLA	5	371.1	18.9	18.0
PLA/BC-*g*-PLA10	BC-*g*-PLA	10	367.6	19.8	18.5

a Note: DSC and TGA data used to assess thermal stability and crystallization behavior are provided in the SI.

#### Morphological analysis of composite film

**Fig. 4:**
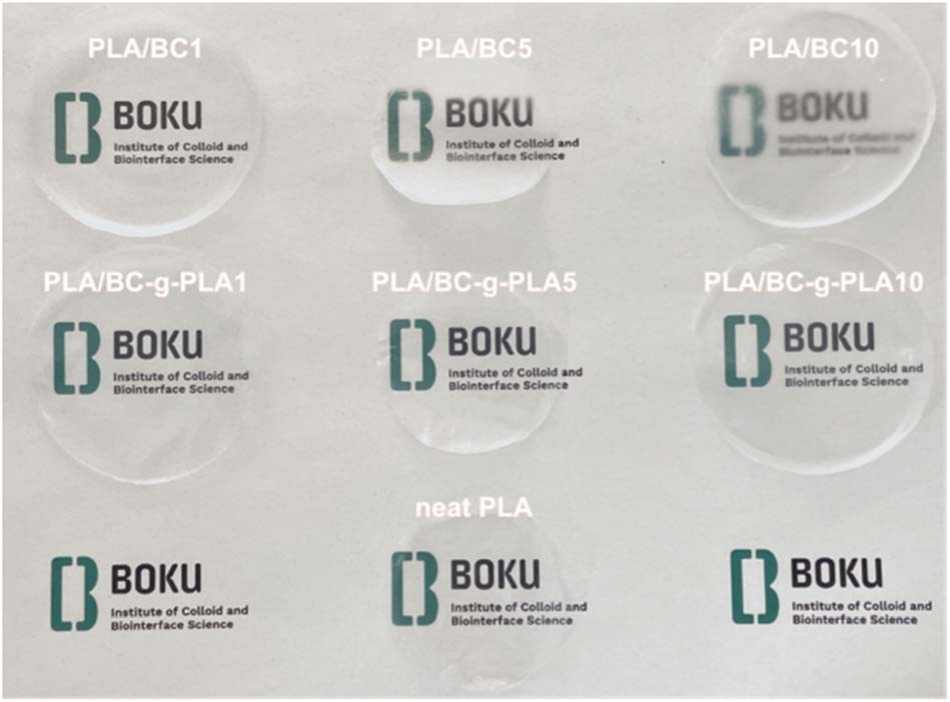
Photographs of PLA composite films containing 0, 1, 5, and 10 wt% of unmodified BC or BC-*g*-PLA. All films were prepared by solvent casting and had a thickness of approximately 60 µm.

Visual observation of the films prepared with unmodified and surface-modified BC fillers at 0, 1, 5, and 10 wt% showed that BC-*g*-PLA improved optical clarity compared to films containing unmodified BC filler (Fig. 4). The transparency of PLA with dispersed BC-*g*-PLA was comparable to that of neat PLA. Additionally, the sample with BC-*g*-PLA filler exhibited a smooth surface, similar to that of neat PLA, whereas the unmodified BC sample had a rough surface. The film containing unmodified BC clearly exhibited poor dispersion, as evidenced by poor transparency and visible agglomerates and surface heterogeneity. Due to the macroscale BC aggregation, SBF-SEM imaging was not performed on these samples. The dispersion states of neat PLA, PLA/BC-*g*-PLA1, PLA/BC-*g*-PLA5, and PLA/BC-*g*-PLA10 were observed using SBF-SEM.

This advanced imaging technique uses an ultrasharp microtome to slice 150 nm-thick layers and generate extremely smooth surfaces for stepwise imaging, thereby enabling visualization of the nanoscale distribution, orientation, and aggregation of the fillers within the bulk of the sample. Such internal structural information cannot be obtained using conventional 2D-SEM, which provides only surface or single-fracture-plane images.

**Fig. 5:**
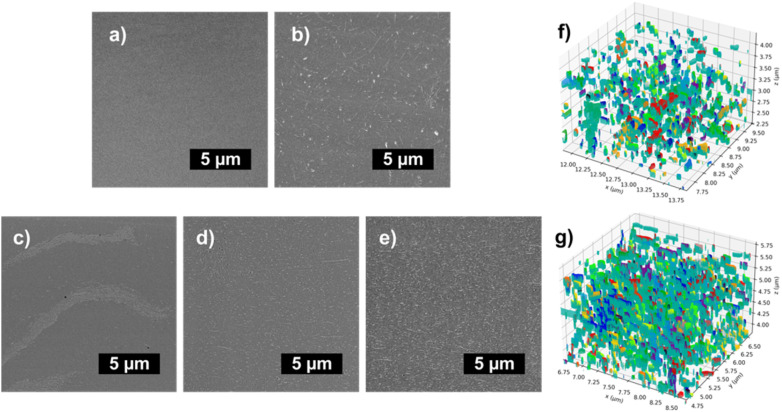
Scanning electron micrographs of cross-sectional surfaces obtained by SBF-SEM: (a) neat PLA film sliced perpendicular to the film plane; (b) PLA/BC-*g*-PLA5 sliced parallel to the film plane; (c)–(e) PLA/BC-*g*-PLA1, PLA/BC-*g*-PLA5, and PLA/BC-*g*-PLA10, respectively, sliced perpendicular to the film plane; (f) and (g) selected subvolumes (2 × 2 × 2 µm^3^) from the 3D reconstructed models of PLA/BC-*g*-PLA5 and PLA/BC-*g*-PLA10, respectively, generated by stitching particles from contrast features from sequential cross-sections (5 × 5 × 3 µm^3^).

Fig. 5 shows SBF-SEM images of neat PLA and composite samples. No discernible features were observed in the neat PLA image, whereas PLA/BC-*g*-PLA5 and PLA/BC-*g*-PLA10 exhibited distinct contrast patterns. The observed features originate from the presence of BC-*g*-PLA fillers. In both composite samples, the fillers are homogeneously dispersed throughout the matrix.

Dispersed nanoparticles exhibit negligible light scattering compared with aggregated particles, as the scattering intensity increases approximately with size as D^6^, thereby preserving film transparency relative to PLA/unmodified BC composites containing aggregated fillers. Fig. 5b shows a cross-sectional image of PLA/BC-*g*-PLA5 cut parallel to the film plane, while Fig. 5d and e show cross-sections of PLA/BC-*g*-PLA5 and PLA/BC-*g*-PLA10, respectively, obtained by cutting perpendicular to the film plane.

These images illustrate the excellent dispersion of BC nanofibers, which were analyzed after 3D reconstruction to have average lengths of ∼0.6 and ∼1 µm, respectively, and an average diameter of ∼140 nm, with large size distributions and aspect ratios of ∼18 and ∼37, respectively. This demonstrates that the BC fibers were both well-dispersed and, to a large degree, remained stretched in the PLA matrix. In contrast, PLA/BC-*g*-PLA1 exhibited noticeable filler aggregates (Fig. 5c). This indicates that uniform nanoscale dispersion had not been fully achieved in this sample. As all forms of aggregation during casting should increase with filler concentration, we attribute the aggregates of BC-*g*-PLA in this sample to insufficient dispersion in chloroform during the solvent-casting process.

To further investigate the spatial distribution and orientation of the fillers, a 3D reconstruction of PLA/BC-*g*-PLA5 and PLA/BC-*g*-PLA10 was performed by stacking a series of SBF-SEM images acquired from slices perpendicular to the film plane (Fig. 5f and g). Based on the imaging contrast, the average filler volume fractions were estimated to be 3.4% for PLA/BC-*g*-PLA5 and 6.2% for PLA/BC-*g*-PLA10, corresponding to ∼58 and ∼80 particles per µm^3^, respectively. The grafted PLA will differ only marginally from the PLA matrix and is therefore unlikely to be distinguishable in SBF-SEM due to a lack of topographical and electron-density contrast. Hence, the values derived from areas with different contrast represent mainly the BC portion of the filler, which accounts for 43 wt% of BC-*g*-PLA according to TGA analysis. Given the approximate density ratio of 1.2 between BC and PLA, the corresponding weight fractions of ∼6 wt% and ∼12 wt% calculated for PLA/BC-*g*-PLA5 and PLA/BC-*g*-PLA10, respectively, are very close to the nominal weight fractions. Given the uncertainty in the border of high contrast observable for densely grafted PLA on the BC nanofibers, the observed proportional increase of the calculated weight fractions of PLA/BC-*g*-PLA5 to PLA/BC-*g*-PLA10 is fully consistent with expectations and supports the interpretation that BC-*g*-PLA is homogeneously dispersed in the PLA matrix.

The differences in observed BC fiber morphology between Fig. 5b, d and e suggest that, within the observed regions, the fillers may exhibit weak preferential alignment. To quantitatively assess filler orientation, the nematic order parameter was calculated for particles containing a sufficient number of voxels to define a directional vector. The coordinate system is defined such that the *z*-axis is parallel to the tensile direction, while the *x*–*y* plane is perpendicular to it. A unit vector describing the particle orientation was determined for each object. Sub-volumes of ∼8 µm^3^ were analyzed throughout the sample to visualize spatial variations in alignment (Fig. S4). The results indicate that no strong global ordering is present and that this is due to weak local alignment rather than large-scale heterogeneity. Globally, both PLA/BC-*g*-PLA5 and PLA/BC-*g*-PLA10 exhibited nematic order parameters of approximately 0.10, with the global director oriented close to the *y*-axis for PLA/BC-*g*-PLA5 and close to the *z*-axis for PLA/BC-*g*-PLA10, while sub-volumes showed locally higher alignment, *e.g.*, near the edge of the sample. These values, corresponding to weak alignment, are consistent with expectations for solvent casting, which can exhibit weak flow and evaporation effects, but are not consistent with large-scale or long-range fiber ordering, as was suggested by Fig. 5.

#### Mechanical properties and fracture mechanism

**Fig. 6:**
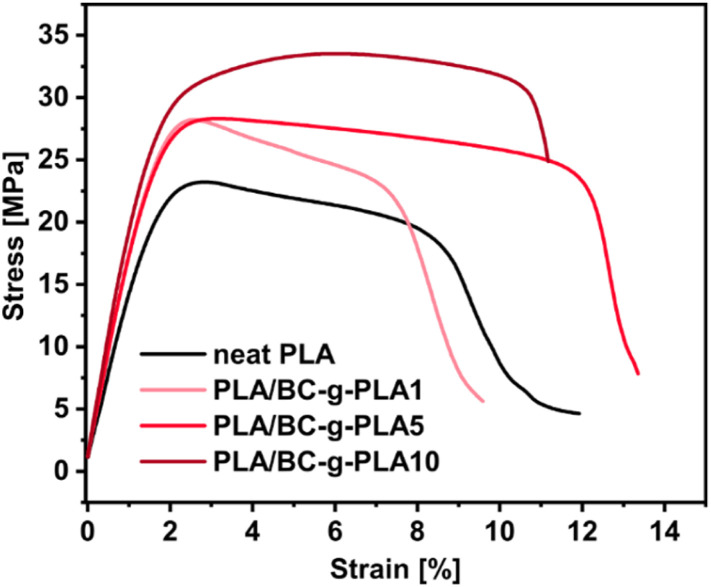
Typical stress–strain curve of neat PLA and PLA composites containing 1, 5, and 10 wt% of BC-*g*-PLA.

The mechanical properties of all samples are summarized in Table 2. Neat PLA was used as the reference material for evaluating the reinforcing effect of BC-*g*-PLA on PLA. PLA/BC-*g*-PLA1, PLA/BC-*g*-PLA5, and PLA/BC-*g*-PLA10 showed increases of 18.6%, 19.2%, and 43.8% in maximum stress; 16.5%, 11.6%, and 20.0% in yield stress; and 23.7%, 26.8%, and 52.4% in Young's modulus, respectively, while no significant difference was observed in elongation at break. As shown in Fig. 6, all samples exhibited a distinct yield point at a strain of approximately 2%, corresponding to the onset of plastic deformation of the PLA matrix. Compared with extrusion-processed PLA systems reported in the literature, the present solvent-cast films exhibit slightly lower tensile strength but higher elongation at break, which is commonly observed for PLA materials prepared *via* solvent casting.^27^

**Table 2:** Mechanical properties of PLA and PLA/BC-*g*-PLA composites. 0.2% offset yield stress (*σ*_y_), maximum stress (*σ*_max_), Young's modulus (*E*), elongation at break (*ε*_break_), and toughness of PLA, PLA/BC-*g*-PLA1, PLA/BC-*g*-PLA5, and PLA/BC-*g*-PLA10^a^

Sample	*σ*_y_ [MPa]	*σ*_max_ [MPa]	*E* [GPa]	*ε*_break_ [%]	Toughness [MJ m^−3^]
PLA	20.4 ± 0.3	23.3 ± 0.4	1.31 ± 0.03	11.7 ± 1.3	2.10 ± 0.28
PLA/BC-*g*-PLA1	23.7 ± 0.1***	27.6 ± 0.1***	1.62 ± 0.01***	10.5 ± 0.6^ns^	2.01 ± 0.15^ns^
PLA/BC-*g*-PLA5	22.7 ± 0.1***	27.8 ± 0.2***	1.66 ± 0.01***	13.7 ± 1.0^ns^	3.13 ± 0.24*
PLA/BC-*g*-PLA10	24.5 ± 0.3***	33.5 ± 0.4***	2.00 ± 0.03***	11.3 ± 1.3^ns^	3.22 ± 0.35*

a Values are reported as mean ± standard deviation (*n* = 5). Statistical significance: *** *p* < 0.001, ** *p* < 0.01, * *p* < 0.05, ns *p* ≥ 0.05, based on *t*-tests comparing each sample to PLA.

In PLA nanocomposite systems, improvements in stiffness and strength are typically accompanied by a reduction in elongation at break (ductility), particularly when rigid nanocellulose fillers are incorporated.^28,29^ A similar trend has also been reported for PLA systems containing OLA-grafted nanocellulose fillers,^21,30–32^ although the number of systematic studies on PLA- or OLA-grafted nanocellulose remains limited.

In contrast, the present PLA/BC-*g*-PLA composites maintained the elongation at break while exhibiting clear increases in Young's modulus and tensile strength.

**Fig. 7:**
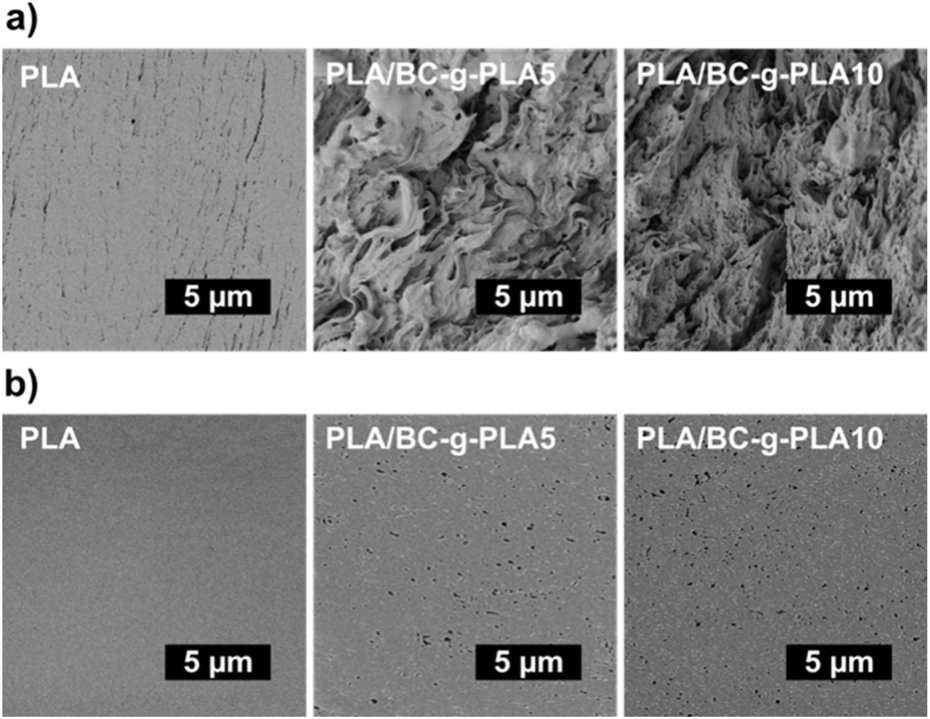
Scanning electron microscope images of (a) the fracture surface after tensile testing, and (b) microtomed surface using SBF-SEM near the fractured region.

In neat PLA, molecular mobility is severely restricted at room temperature because the glass transition temperature of PLA exceeds ambient temperature. As a result, PLA typically exhibits brittle fracture with limited energy dissipation.^33^ The fracture surface of neat PLA was smooth and featureless, characteristic of brittle cleavage (Fig. 7a).

The addition of PLA-grafted BC fillers resulted in a significant improvement in mechanical properties. This was enabled by the surface modification, which reduced effective particle attraction and enhanced the affinity between BC and PLA, resulting in homogeneous filler dispersion within the matrix. It is well established that filler agglomeration can lead to stress concentration and deterioration of mechanical properties.^34–36^ The homogeneous dispersion achieved here effectively avoided such issues. Enhanced interfacial adhesion also enabled efficient stress transfer across the filler–matrix interface. Interfacial debonding and filler pull-out are typical failure modes when interfacial strength is insufficient,^37^ resulting not only in the loss of load-bearing capacity but also in the initiation of matrix cracking. SEM observations of the fracture surfaces revealed no evidence of catastrophic debonding or pull-out (Fig. 7a). The fracture surfaces of PLA/BC-*g*-PLA10 and PLA/BC-*g*-PLA5 showed BC fillers bridging across the fracture plane, demonstrating that interfacial adhesion was preserved even during fracture and that the fillers effectively supported matrix deformation. These results suggest that the improved interfacial bonding contributed to the increased Young's modulus and yield stress observed up to the yield point, consistent with previous studies on PLA–filler composites.^38^

In the present study, no significant reduction in elongation at break was observed despite the improvement in Young's modulus and maximum and yield stress. To elucidate this behavior, the deformation mechanism beyond the yield point was examined. SEM analysis of cross-sections of the region near the fracture surface revealed numerous nanoscale voids within the PLA bulk material in the PLA/BC-*g*-PLA composites (Fig. 7b). The BC particles and voids comprised ∼5% and ∼3% of the area for PLA/BC-*g*-PLA5 and ∼10.6% and ∼1.4% of the area for PLA/BC-*g*-PLA10, respectively, showing excellent correspondence with the expected fractions of BC. Spatial association analysis of the distribution of fibers and voids resulted in a mean *Z*-score of −8.7 (*p* < 0.001) for all voids but a *Z*-score of 13.9 (*p* > 0.999) for large expanding voids (>50 pixels) for PLA/BC-*g*-PLA10. For PLA/BC-*g*-PLA5, we observed a mean *Z*-score of −47.1 (*p* < 0.001) for all voids and a *Z*-score of −5.5 (*p* < 0.001) for large voids (>50 pixels). A positive *Z*-score indicates avoidance, while a negative *Z*-score indicates co-localization, and a high *p*-value indicates significant avoidance, while a low *p*-value indicates significant co-localization. Further, 74% of all voids and 92% of large voids are in close proximity to a particle for PLA/BC-*g*-PLA5, *i.e.*, essentially overlapping, and 71% of all voids and 91% of all large voids are close to a particle for PLA/BC-*g*-PLA10. These results indicate that even for modified particles, debonding overall tends to occur near the rigid nanofillers. They strongly suggest a mechanism explaining why improved stiffness did not result in reduced elongation at break in our nanocomposites. The significant spatial clustering of nano-voids at the PLA/BC-*g*-PLA interfaces confirms widespread, albeit small, interfacial debonding. This extensive localized cavitation safely absorbs energy, relieves stress, and promotes matrix shear yielding, allowing the material to maintain high strain levels. We presume that, in contrast to unmodified fillers, the BC-*g*-PLA avoids catastrophic crack propagation due to the strong bonding to the matrix despite the large stiffness mismatch between PLA and BC. Hence, many shear-yielding zones can develop while the BC still bears the load.

**Fig. 8:**
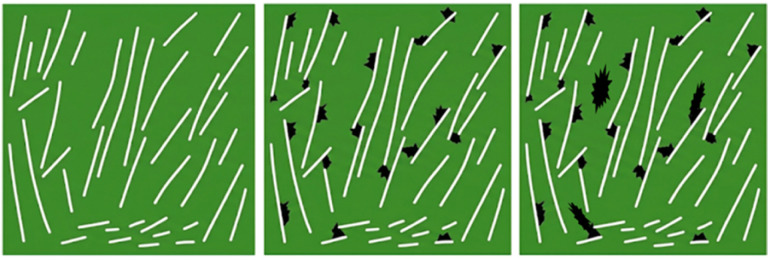
Proposed reinforcement mechanism in PLA/BC-*g*-PLA composites.

Growth and coalescence of voids at large strains can ultimately lead to catastrophic fracture in bulk polymers. In this context, the results for PLA/BC-*g*-PLA10, which is very dense with nanocellulose, are interesting. Here, large voids that appear to be expanding toward catastrophic failure were found to be statistically far from nanoparticles (*Z* = 13.9, *p* = 1.000), despite a high fraction of them being in contact with nanoparticles. For PLA/BC-*g*-PLA5, the *Z*-value was negative for large voids, but with a much lower magnitude than for all (small) voids. This, together with the fact that >90% of large voids in both samples are in contact with a nanofiber, indicates that large voids preferentially grow in large PLA regions and suggests that the nanofibers can act as effective crack arrestors, suppressing the propagation of voids into full-fracture cracks. As a result, the rapid reduction in the effective load-bearing cross-section was suppressed, allowing the material to retain load-bearing capacity during further deformation (Fig. 8). Macroscopically, this behavior appeared as a reduced post-yield stress drop or, in some cases, as a continued stress increase with a lower slope in the stress–strain curve (*cf.*Fig. 6).

We interpret these results as follows: the changes in the slope of the stress–strain curve at higher strains are initially due to local debonding at the PLA/nanofiller interface, dissipating some of the energy from the increased load, until large void formation and subsequent catastrophic crack formation take over. The apparent high strength of the PLA/BC-*g*-PLA interface suggests, in analogy with the simulations, that attractive interactions between the filler and the bulk material can lead to cohesive fracture through voids within the bulk.^39^ As a result of these combined effects, PLA/BC-*g*-PLA10 exhibited a substantial enhancement in toughness, with the area under the stress–strain curve increasing by approximately 53% compared with neat PLA.

PLA/BC-*g*-PLA5 also showed a significant improvement in mechanical properties, but not as much as PLA/BC-*g*-PLA10, and it did not show as clear indications of a continued stress increase in the stress–strain curve as PLA/BC-*g*-PLA10. This is likely due to the lower filler content, where also large propagating voids were predominantly observed close to nanofillers.

Although such analyses are uncertain from 2D images, as particles orthogonal to the imaging plane are not part of the analysis, it is notable that the alignment of particles large enough in the imaging plane to show significant anisotropy (∼10% of all particles) showed low to intermediate nematic order (*S* = 0.41 and *S* = 0.26 for PLA/BC-*g*-PLA5 and PLA/BC-*g*-PLA10, respectively), but nematic coupling parameters of 0.99 and 0.71 for PLA/BC-*g*-PLA5 and PLA/BC-*g*-PLA10, respectively, with the voids, which unsurprisingly showed higher nematic order than the particles. This result suggests that voids are indeed preferentially growing parallel to the nanofibers because the fibers hinder their propagation.

PLA/BC-*g*-PLA1 and PLA/BC-*g*-PLA5 showed comparable improvements in Young's modulus and maximum stress, while the yield stress of PLA/BC-*g*-PLA1 was slightly higher. However, the post-yield behavior of PLA/BC-*g*-PLA1 was nearly identical to that of neat PLA, resulting in no improvement in toughness compared to neat PLA, whereas PLA/BC-*g*-PLA5 displayed a toughness similar to PLA/BC-*g*-PLA10. As noted above, uniform filler dispersion was not achieved in PLA/BC-*g*-PLA1, which likely led to localized stress concentrations and insufficient suppression of void growth and coalescence. As expected, this increased the yield stress but reduced the ductility compared to neat PLA.

Overall, the mechanical behavior of the PLA/BC-*g*-PLA composites can be understood as the result of two distinct contributing mechanisms operating before and after the yield point. Prior to yielding, the conventional reinforcement effect arising from the incorporation of rigid fillers was effectively manifested, leading to increases in Young's modulus and yield stress. Beyond the yield point, nanoscale voids were growing primarily within the PLA bulk; however, their coalescence and growth were obstructed by the reinforcing effect of the PLA-integrated BC fillers. As a result, the material retained its load-bearing capacity during further deformation, thereby contributing to the simultaneous improvement in strength and ductility. These reinforcing mechanisms were enabled by the uniform dispersion and strong interfacial affinity achieved through PLA surface modification.

#### Water vapor transmission rate (WVTR)

**Fig. 9:**
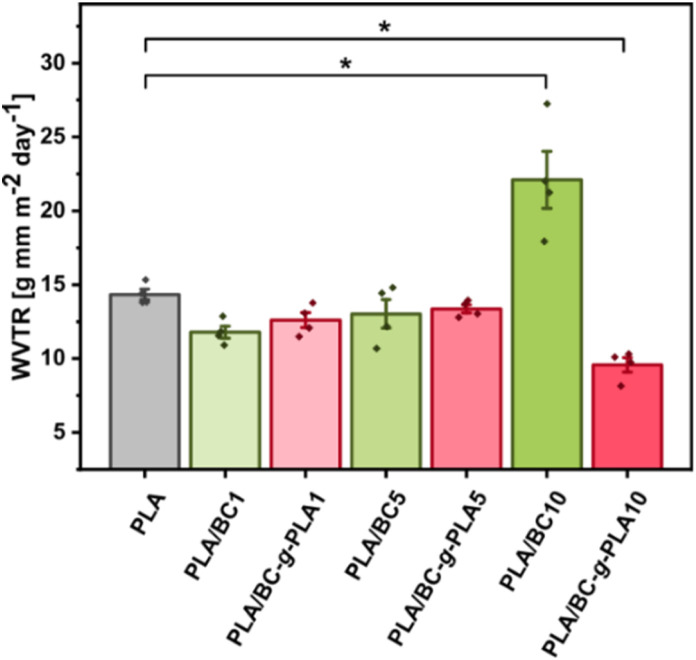
Comparison of water vapor permeabilities in PLA films with 0, 1, 5, and 10 wt% BC or BC-*g*-PLA. All films had a thickness of approximately 60 µm. (*p < 0.05 *vs.* neat PLA).

The WVTR of each film was measured to evaluate its gas barrier properties. The results are presented in Fig. 9. A significant change in WVTR was observed only in the films containing 10 wt% of unmodified BC or surface-modified BC. The incorporation of 10 wt% unmodified BC led to a significant increase in WVTR, likely due to poor filler dispersion, which resulted in surface irregularities and thickness inhomogeneities in the film. The effect became more pronounced with increasing filler content and was particularly severe in PLA/BC10. These morphological defects create shorter diffusion paths and potential permeation hotspots, thereby facilitating vapor transmission. In addition, the intrinsically higher water permeability of cellulose compared to PLA may also contribute to the increased WVTR in PLA/BC10.

In contrast, the addition of 10 wt% surface-modified BC significantly reduced the WVTR. This improvement is likely due to the more homogeneous dispersion of BC-*g*-PLA and improved interfacial compatibility with the PLA matrix. The densely grafted PLA chains are expected to strongly bond the BC surface to the surrounding PLA, effectively keeping the fibers well embedded and surrounded by a relatively low-permeability PLA-rich interfacial region. Homogeneously dispersed BC-*g*-PLA fillers may therefore act as physical barriers that elongate and complicate the diffusion pathways.^40^ Under such conditions, the tortuosity effect becomes more plausible despite the intrinsically higher permeability of cellulose.

However, the remaining samples, particularly those with lower filler content, exhibit no clear or systematic trend. Although some previous studies have reported reductions in gas permeability at lower filler loadings,^41,42^ such discrepancies are likely attributable to differences in filler type, morphology, degree of dispersion, processing conditions, and possible changes in the bulk PLA phase, such as variations in crystallinity.

#### Biodegradability

**Fig. 10:**
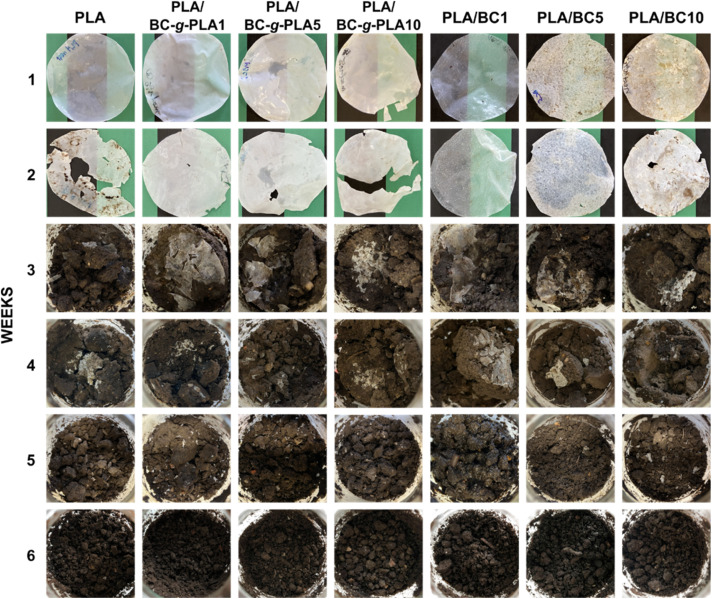
Photographic record of the biodegradation of PLA-based films over a 6-week composting test. From week 4 onward, the soil was carefully displaced to document the *in situ* condition of the films due to their increased brittleness.

**Fig. 11:**
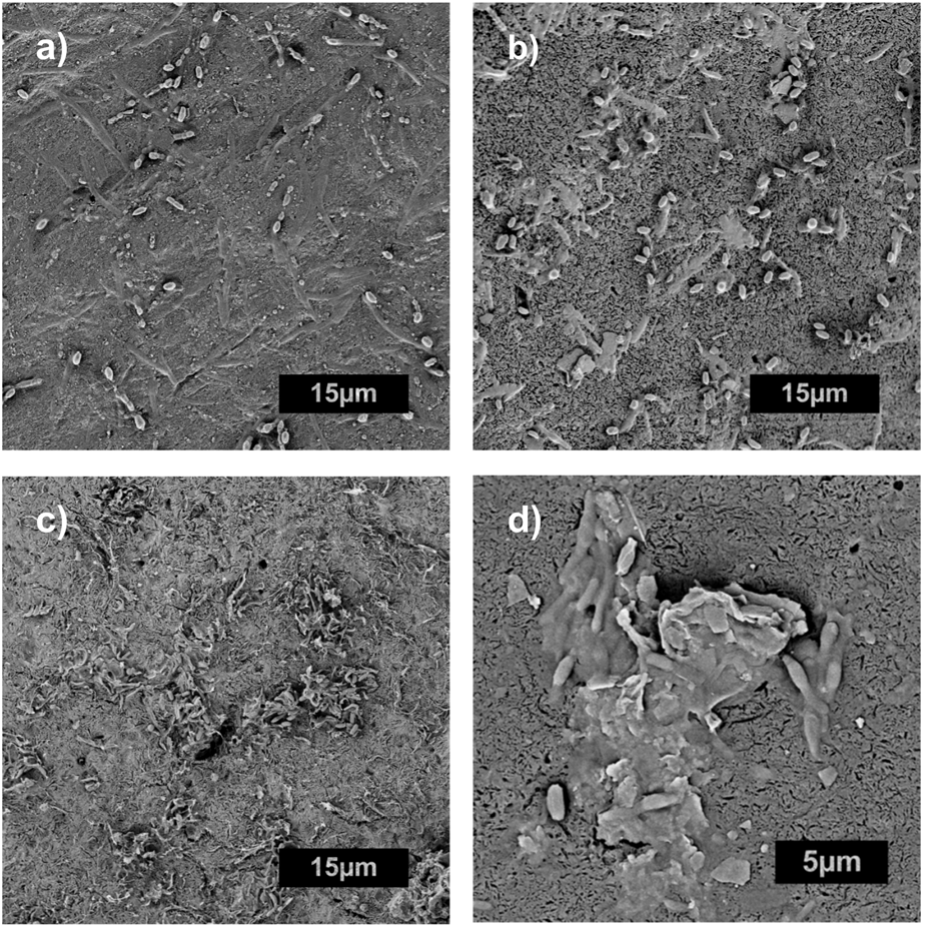
SEM images of the film surfaces at week 3 of biodegradation testing. (a) PLA, (b) PLA/BC-*g*-PLA5, (c) PLA/BC5, and (d) bacterial colony formation covered by extracellular polymeric substances on PLA/BC-*g*-PLA.

The biodegradability of each film was qualitatively assessed by burying it in compost maintained at 60 °C, with the degradation process documented photographically (Fig. 10). After approximately three weeks, none of the samples retained their original shape. The surfaces of these degraded samples were examined by SEM (Fig. 11a–c), which revealed bacterial adhesion on all sample types. In some areas, bacteria were observed forming colonies and appearing to be embedded in extracellular polymeric substances (Fig. 11d).

By the sixth week, the films could no longer be visually identified in the compost. Based on visual and morphological observations, no noticeable differences in degradation behavior were observed among the samples, regardless of filler type or content. These results suggest that incorporating BC or BC-*g*-PLA does not impair PLA biodegradability under composting conditions.

## Conclusion

In this study, PLA-grafted bacterial cellulose (BC-*g*-PLA) was employed to address the strength–ductility trade-off commonly observed in PLA composites. Grafting PLA chains onto BC significantly improved interfacial compatibility with the PLA matrix, enabling homogeneous nanoscale dispersion. Consequently, the composites exhibited higher Young's moduli and tensile strengths than neat PLA while maintaining ductility, resulting in a significant increase in toughness. These improvements increased monotonically with increasing mass loading of the modified nanofiller up to 10 wt%, the highest loading tested.

Three-dimensional analysis by SBF-SEM further revealed that the uniformly dispersed BC-*g*-PLA fibers exhibited weak, preferential alignment over macroscopic distances, with locally higher nematic ordering. Further analysis of composites after mechanical testing revealed nanocavitation on the fibers and void growth confined to align with the fibers through the bulk, leading to obstructed matrix shear yielding as the cause for the improved properties. This is consistent with the continued stress increase observed in the well-dispersed 10 wt% BC-*g*-PLA composites after the yield strength is reached, for which these structural features were most pronounced.

Incorporation of 10 wt% BC-*g*-PLA reduced water vapor transmission, while biodegradability under composting conditions remained unaffected by the presence of either BC or BC-*g*-PLA.

In summary, this work demonstrates that combining interfacial design through PLA grafting with controlled nanoscale dispersion is an effective strategy for mitigating the strength–ductility trade-off in PLA composites, providing fundamental insights for the development of high-performance and biodegradable polymer materials.

## Author contributions

Yuuki Takatsuna and Ronald Zirbs conceived the ideas. Yuuki Takatsuna conducted material preparation, data collection, and evaluation, and wrote the first draft of the manuscript. Ronald Zirbs supervised the project, and Erik Reimhult co-supervised it and performed the SBF-SEM data analysis. All authors contributed to the writing of the manuscript.

## Conflicts of interest

The authors have no competing interests to declare that are relevant to the content of this article.

## Supplementary Material

RA-OLF-D6RA04355K-s001

## Data Availability

The data supporting this article have been included in the manuscript or as part of the supplementary information (SI). Additional raw data, such as full image stacks that are too large and impractical to include in the submission, will be made available by the authors upon request. After the PhD student project is completed, all data and additional scripts will be made available in an open-access repository (Zenodo for standard data, and a local repository for image stacks too large for Zenodo and similar general repositories). Supplementary information is available. See DOI: https://doi.org/10.1039/d6ra04355k.
