# Supplementary material for: Grafting of bacterial cellulose nanofibers with polylactic acid (PLA) enables homogeneous dispersion in PLA and improved strength and stiffness without loss of ductility
Source: RSC Adv. 2026 Jul 10;16(37):39604–14. doi: 10.1039/d6ra04355k (PMC13353058; doi:10.1039/d6ra04355k)
Supplement: Supplementary file 1 [file RA-016-D6RA04355K-s001.pdf]

Supplementary Information (SI) for RSC Advances.
This journal is © The Royal Society of Chemistry 2026

Grafting of bacterial cellulose nanofibers with polylactic acid (PLA) enables homogeneous dispersion in PLA and improved strength and stiffness without loss of ductility

Yuuki Takatsuna, Erik Reimhult and Ronald Zirbs**

Institute of Colloid and Biointerface Science, BOKU University, Muthgasse 11/II, A-1190 Vienna, Austria

*e-mail: erik.reimhult@boku.ac.at, ronald.zirbs@boku.ac.at

Number of pages: 13

Number of figures: 8

Number of tables: 8

Transmission electron microscopy (TEM) measurements

TEM images were recorded with an FEI Tecnai G2 (FEI Europe B.V., Austria) with 160 kV acceleration voltage. cBC-g-PLA was dispersed in dichloromethane, sonicated for 10 minutes. A small amount of the dispersion was dropped onto the carbon grids and dried under ambient conditions.

TEM results

Fig. S1 shows the fibrous morphology of BC-g-PLA observed by TEM, which is consistent with the morphology observed in the SEM images.

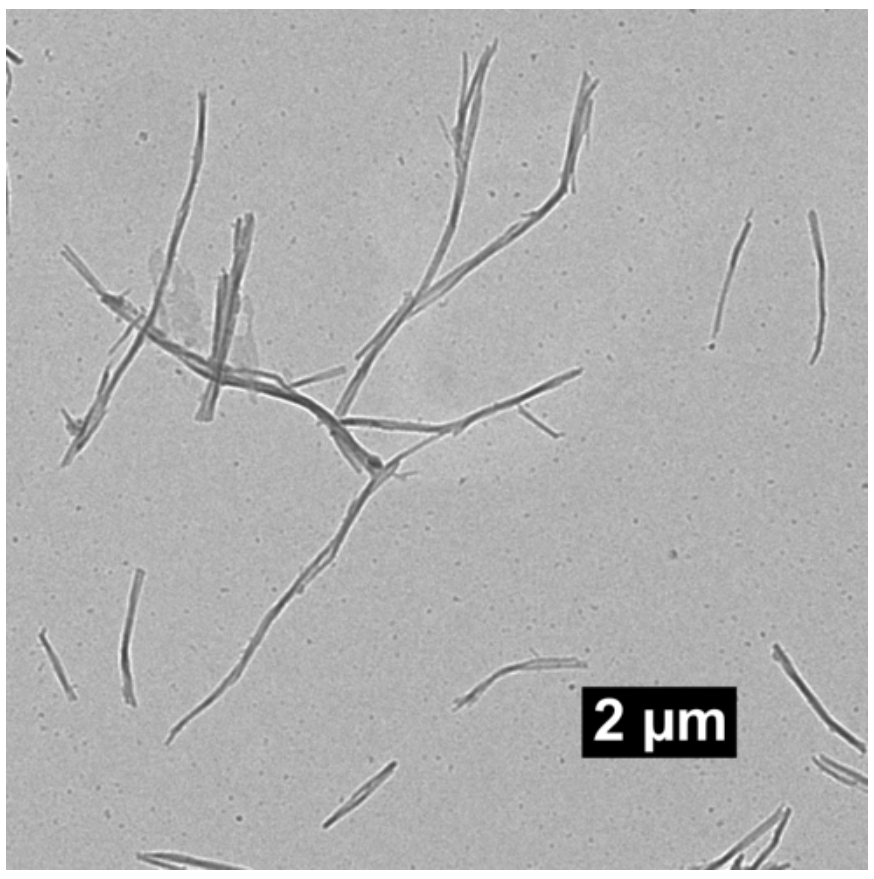

Fig. S1 Transmission electron microscopy image of BC-g-PLA

DSC results

Fig. S2 shows the DSC curves of each sample during the first and second heating cycles, and the crystallinity of the samples was calculated. In both heating cycles, the addition of unmodified BC resulted in higher crystallinity, which is likely due to its surface promoting heterogeneous nucleation during cooling. In contrast, the presence of a distinct cold crystallization peak was not observed for the unmodified BC composites, indicating that most crystallization had already taken place during the cooling stage.

PLA composites containing surface-modified BC exhibited a different behavior. Because the grafted PLA chains render the filler surface chemically like the PLA matrix, the nucleating efficiency of the BC surface is likely reduced. As a result, composites with BC-g-PLA showed a clear cold crystallization peak during the second heating cycle, suggesting that a larger fraction of amorphous PLA remained after cooling. Consequently, the overall crystallinity of these composites was lower than that of the unmodified BC-filled samples.

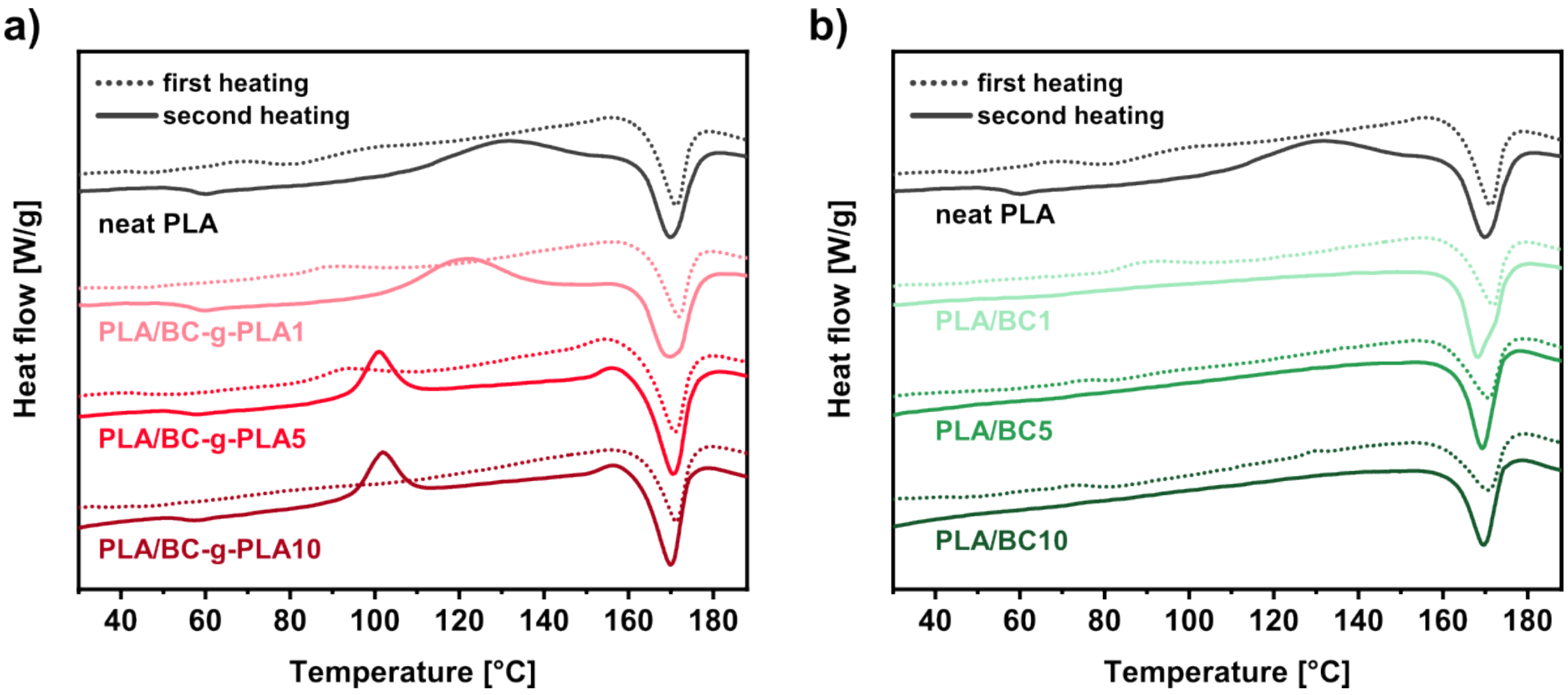

Fig. S2 DSC heating curves of PLA with 1, 5 and 10wt% of a) BC-g-PLA and b) unmodified BC.

Differential thermogravimetric analysis measurements

The thermal stability of each sample was evaluated by comparing the maximum degradation temperature (T_{max}) obtained from TGA analysis. The obtained DTG curves are shown in Fig. 5a. All samples exhibited nearly identical T_{max}, indicating that the incorporation of either unmodified BC or BC-g-PLA did not noticeably affect the thermal stability of the PLA matrix.

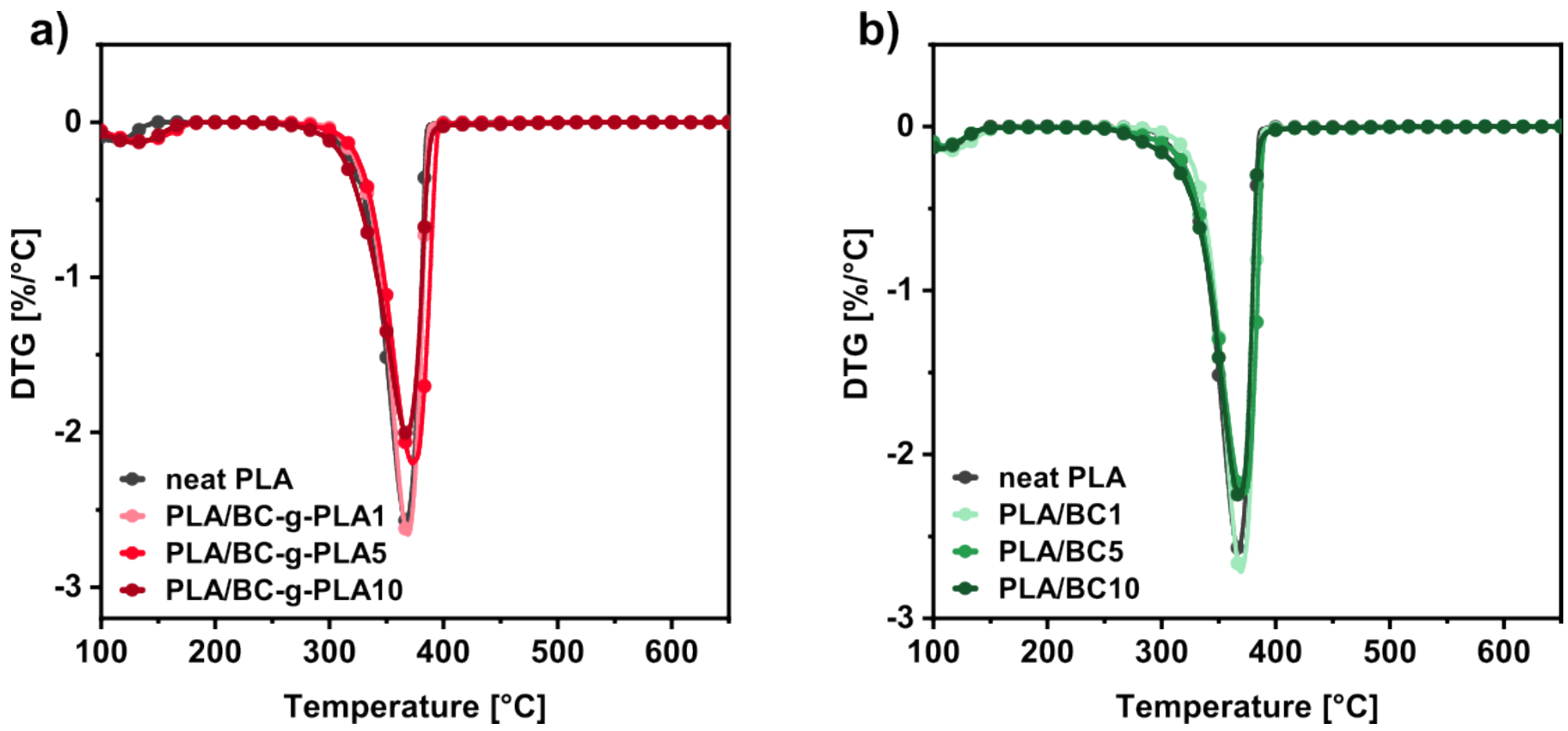

Fig. S3 DTG curves obtained from TGA analysis of PLA with 1, 5 and 10wt% of a) BC-g-PLA and b) unmodified BC.

Stress-strain curves

shows the stress–strain curves of neat PLA and PLA composites containing 1, 5, and 10 wt% of BC-g-PLA. A clear trend is observed, i.e., higher filler contents result in increased yield stress, Young's modulus, and maximum stress.

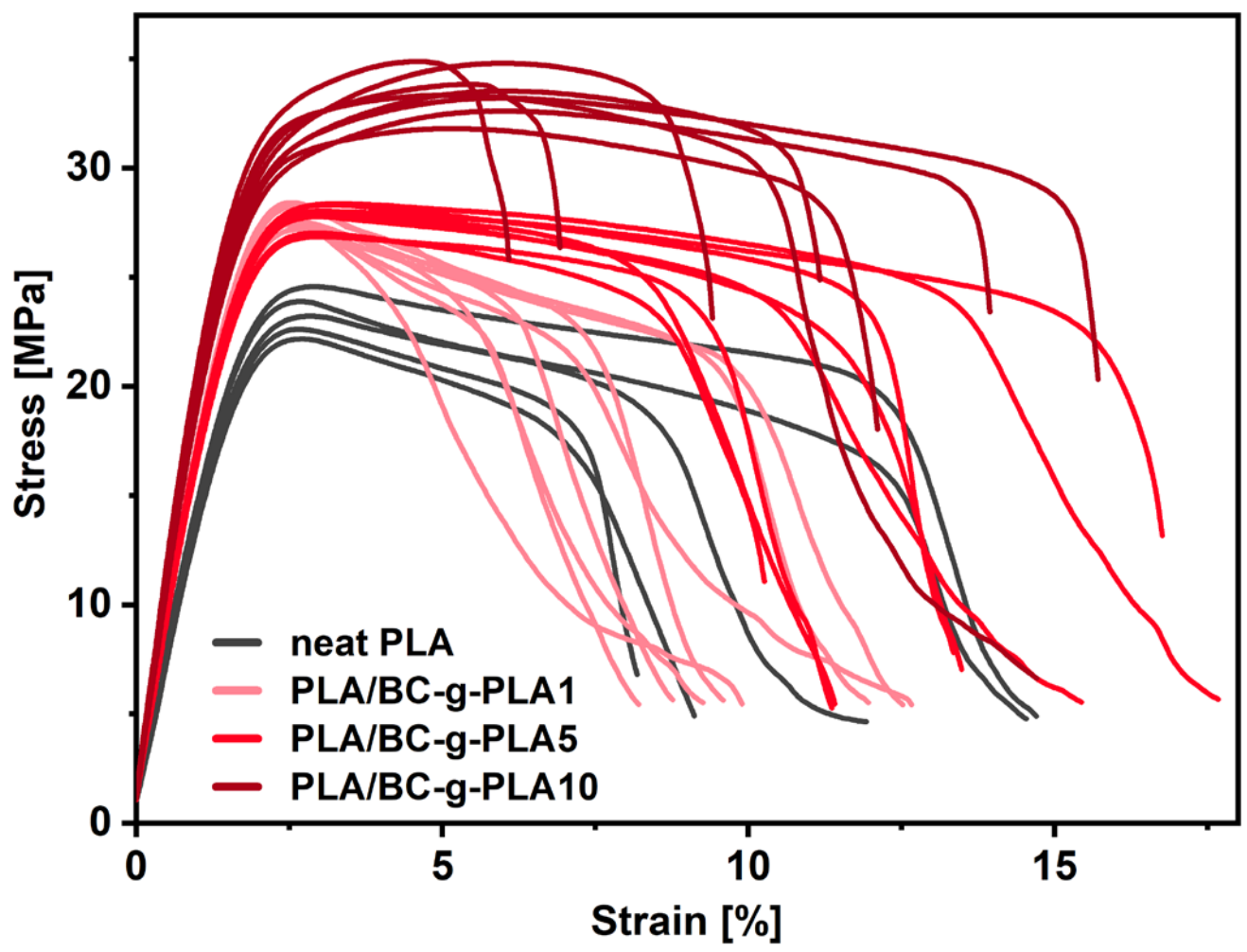

Fig. S4 Stress–strain curves of neat PLA and PLA composites containing 1, 5, and 10 wt% of BC-g-PLA.

Image analysis of Amira VolumeScope serial-blockface SEM data

The 3D tomographic data (AmiraMesh or HDF5 format) was imported and parsed into a 3D NumPy array. Subsequently, individual particles were identified and uniquely indexed using a 3D connected-component labeling algorithm via skimage.measure.label. Basic geometric properties for each identified particle were extracted utilizing skimage.measure.regionprops. Sphericity was calculated by generating a 3D mesh of each particle using the Marching Cubes algorithm (skimage.measure.marching_cubes) to determine the exact surface area, which was then compared to the surface area of a perfect sphere of equivalent volume. Solidity was computed as the ratio of the particle's volume to its convex hull volume.

To analyze the true morphology of bent or tortuous fibers, a thickness-invariant contour analysis was implemented. Each particle was reduced to a 1D medial axis using skimage.morphology.skeletonize. A topological graph of the skeleton was constructed using the networkx library, where nodes represented skeleton voxels and edge weights represented the Euclidean distances between 26-connected neighbors, computed via scipy.spatial.cKDTree. The true contour length was determined by finding the longest geodesic path through this graph using Dijkstra's algorithm (networkx.single_source_dijkstra). Simultaneously, the 3D Euclidean Distance Transform (EDT) of the entire void space was computed using scipy.ndimage.distance_transform_edt. The mean contour diameter of each particle was derived by extracting and averaging the EDT radii exactly along its longest skeletal path.

To robustly determine the primary orientation and physical dimensions of each particle, Principal Component Analysis (PCA) was performed on the point cloud of each labeled particle's constituent voxel coordinates for particles with a volume larger than 10 voxels. The 3x3 covariance matrix of the physically scaled, mean-centered coordinates was diagonalized using numpy.linalg.eigh. The eigenvector corresponding to the largest eigenvalue defined the particle's primary orientation vector (local director). The physical Length and PCA-derived Diameter were

calculated by projecting the voxel coordinates onto these principal axes to define the physical bounding envelope of the object.

The macroscopic alignment of the particulate system was quantified using the Q-tensor approach. A global orientational Q-tensor was constructed from the valid individual orientation vectors. By diagonalizing this tensor, the global director was identified as the eigenvector associated with the largest positive eigenvalue, which corresponds to the global scalar nematic order parameter, S. To evaluate short-range alignment, a local order parameter was calculated for each particle by averaging the second Legendre polynomial of the angles between the reference particle and its 15 nearest spatial neighbors, identified using a Kd tree spatial index (scipy.spatial.cKDTree).

Table S1 Parameters used for the fitting of the PLA/BC-*g*-PLA5 Amira data using the custom-written Python script Amira_Data_Analyzer.

Parameter	Value
Voxel Size X (um)	0.0090
Voxel Size Y (um)	0.0090
Voxel Size Z (um)	0.1178
Total Volume (um^3)	2833.7248
Number Density (part/um^3)	58.4111
Volume Fraction	0.0340
Number of Particles	165521
Mean Particle Volume (um^3)	0.0006
Calculated Monomer Diam (um)	0.1417
Calculated Monomer Vol (um^3)	0.0028
Mean Aggregation Number	0.2049

Table S2 Output parameters from the fitting of the PLA/BC-*g*-PLA5 Amira data using the custom-written Python script Amira_Data_Analyzer.

Parameter	Value
Mean Sphericity	0.0000
Mean Solidity	0.9175
Mean Radius of Gyration (um)	0.0324
Mean Length PCA (um)	0.1386
Mean Diameter PCA (um)	0.0769
Mean PCA Aspect Ratio (L/T)	2.1855
Mean Flatness Ratio	1.5691
Mean Contour Length (um)	0.4916
Mean Contour Diam (um)	0.1417
Mean Contour Aspect Ratio (L/D)	18.2741
Mean Orientation (Deg to Z)	50.1124
Mean Azimuthal (Deg in XY)	64.1256
Nearest Neighbor Dist (um)	0.1672
Global Nematic Order S	0.2021
Mean Local Order S	0.0725
Director X	0.2383
Director Y	-0.9708
Director Z	0.0269

Table S3 Parameters used for the fitting of the PLA/BC-*g*-PLA10 Amira data using the custom-written Python script Amira_Data_Analyzer.

Parameter	Value
Voxel Size X (um)	0.0090
Voxel Size Y (um)	0.0090
Voxel Size Z (um)	0.1477
Total Volume (um^3)	1662.9132
Number Density (part/um^3)	80.6314
Volume Fraction	0.0617
Number of Particles	134083
Mean Particle Volume (um^3)	0.0008
Calculated Monomer Diam (um)	0.1590
Calculated Monomer Vol (um^3)	0.0040
Mean Aggregation Number	0.1906

Table S4 Output parameters from the fitting of the PLA/BC-*g*-PLA10 Amira data using the custom-written Python script Amira_Data_Analyzer.

Parameter	Value
Mean Sphericity	0.0000
Mean Solidity	0.8944
Mean Radius of Gyration (um)	0.0386
Mean Length PCA (um)	0.1647
Mean Diameter PCA (um)	0.0922
Mean PCA Aspect Ratio (L/T)	2.0867
Mean Flatness Ratio	1.5041
Mean Contour Length (um)	0.9155
Mean Contour Diam (um)	0.1590
Mean Contour Aspect Ratio (L/D)	36.5565
Mean Orientation (Deg to Z)	51.1716
Mean Azimuthal (Deg in XY)	63.3184
Nearest Neighbor Dist (um)	0.1319
Global Nematic Order S	0.2808
Mean Local Order S	0.0951
Director X	-0.9997
Director Y	-0.0193
Director Z	-0.0155

Image analysis of voids and particles in cross-section SEM images

Image analysis of the shape, orientation, and mutual distances of voids and particles after mechanical testing was performed using a custom-written Python script to batch-process the raw TIFF images.

Briefly, voids were segmented using a Matrix-Capping technique combined with a Kneedle algorithm to precisely identify the intersection between the dark void plateau and the polymer matrix background Gaussian peak in the intensity histogram. Particles were identified using local adaptive mean thresholding to compensate for uneven illumination and charging artifacts. For each, a binary mask was created. Morphological opening (erosion followed by dilation) was applied to the void and particle binary masks, respectively, to eliminate single-pixel salt-and-pepper noise while preserving feature boundaries. The resulting masks were processed using OpenCV's connectedComponentsWithStats and Suzuki's topological structural analysis algorithm (cv2.findContours) to clump connected pixels into voids and particles and to draw a continuous boundary line around each object, which was used for overlap and further object

parameter calculations.

SEM charging and increased electron extraction at sharp features create thin, bright rims around deep voids, which can be falsely classified as particles by automated intensity-based image analysis. Hence, a 1-pixel-thick boundary shell is generated around each detected particle and then compared with the 2-pixel-dilated void contact zones. Particles exceeding the maximum contact ratio of 40% of their boundaries overlapping with the contact zones of a void were rejected as charging artifacts, thereby eliminating only thin rims.

Comprehensive measurements are extracted for all morphological features, including area equivalent diameter, Feret's diameter, and shape factors (Circularity, Convexity, Solidity). The 2D Q-tensor for each object with an area larger than 50 pixels (thereby enabling meaningful size and anisotropy to be assigned in the image plane) was derived from the central moments. It was used to calculate the nematic order parameter (S, 1 implies perfect nematic order) and global director angle (θ) for voids and particles, respectively, for each image. An inter-species (void and particle) coupling parameter ($S_{coupled} = \cos(2\Delta\theta)$) was calculated to determine the relative orientation between voids and particles. Interpretation: $S_{coupled} = 1$ implies perfect parallel alignment, $S_{coupled} \approx 0$ indicates random or independent orientations, and $S_{coupled} = -1$ signifies perfect orthogonal (perpendicular) alignment. These values were statistically summarized for all images of a sample. A null-model spatial Monte-Carlo simulation determined whether voids and particles clustered, i.e, whether they were closer or farther together than would be expected from a random distribution. The Jump Flooding Algorithm (JFA) computed continuous distance maps to find the closest distance of every void boundary to a particle boundary. The void mask was then randomly rolled across the image one thousand times to build a random distribution of mean edge-to-edge distances, against which the observed distances in the original images were statistically compared (Z-score and p-value), and then averaged into a score for all the sum of all images of a sample using Stouffer's formula. Additionally, a 'touching test' was performed. A discrete interaction zone (halo) was dilated around particles in the binary masks. An algorithm computed the percentage of voids that physically intersect this halo, providing an additional measure of whether voids connected to particles.

The parameters used for the analysis and listed in Tables S5 and S7 are explained shortly below: Here is a dictionary detailing each parameter from the Lab Log table.

Thresholding Method: The algorithms used for feature segmentation. Voids are identified by capping the matrix intensity using a Kneedle algorithm and applying a strict difference threshold, while particles are segmented using a localized adaptive mean threshold to account for uneven illumination.

Min Void Area (px): The minimum size requirement in pixels for a dark region to be classified as a valid void. This filters out single-pixel noise and negligible imaging artifacts in the matrix.

Min Particle Area (px): The minimum size requirement in pixels for a bright region to be classified as a valid nanoparticle. This removes noise generated by isolated charging artifacts.

Large Void Threshold (px): The size threshold in pixels used to classify “large” voids (e.g., structural cracks or bulk-expanding cavities) from small tears.

Adaptive Block Size (px): The size of the local sliding window used to calculate the background mean during particle segmentation. A larger block size captures more global illumination trends, while a smaller size is more sensitive to highly localized charging gradients.

Matrix Cap Multiplier: A scalar applied to the calculated histogram intersection (the 'knee' between the void plateau and matrix peak) to establish the upper intensity limit for the bulk polymer matrix. This standardizes the background intensity prior to void extraction.

Void Depth Threshold: The exact grayscale intensity depth below the capped matrix baseline that a pixel must reach to be classified as a void. Lower values increase the algorithm's sensitivity to shallow surface depressions.

Adaptive C Particles: The constant value subtracted from the local background mean during adaptive thresholding. A pixel must be this much brighter than its surrounding neighborhood to be positively classified as a nanoparticle.

Morphological Iterations: The number of times morphological opening (erosion followed by dilation) is applied to the raw particle masks. This cleans up isolated noise pixels while preserving the shape and mass of genuine particles.

Interaction Zone Radius (px): The radius of the morphological dilation applied around each nanoparticle to define an immediate contact or adjacency halo. Voids overlapping this zone are considered to be physically touching or directly adjacent to a particle.

Number of Monte Carlo Simulations: The number of spatial randomizations performed to generate the null model for the edge-to-edge distance distribution. This is used to calculate the statistical significance (Z-score and p-value) of void-particle spatial clustering.

Max Allowed Overlap Ratio: The maximum permissible area overlap fraction between void masks and particle masks during the Monte Carlo randomizations. This enforces a physical-reality constraint on the simulation, preventing the random placement of large voids predominantly atop solid nanoparticles, while allowing for overlapping borders and for voids and particles to overlap in 3D in a 2D projection.

Nematic Exclusion Size (px): The minimum area requirement for a feature to be included in the orientational (nematic) tensor analysis. This prevents tiny features lacking a meaningful longitudinal axis from diluting the global alignment statistics.

Max Void Contact Ratio (Rim Rejection): The maximum allowable percentage of a detected particle's perimeter that can intersect with a dilated void boundary. Particles exceeding this ratio are actively rejected as false positives caused by bright electron charging artifacts commonly observed around the rims of deep voids.

Table S5 Parameters used for the fitting of the PLA/BC-*g*-PLA5 cross-section images using the custom-written Python script GPU_Void-Particle_Analyzer.

**Parameter**	**Value**
Thresholding Method	Matrix-Capped Difference (Voids) + Adaptive (Particles)
Min Void Area (px)	2
Min Particle Area (px)	11
Large Void Threshold (px)	50
Adaptive Block Size (px)	111
Matrix Cap Multiplier	1.1
Void Depth Threshold	1
Adaptive C Particles	11
Morphological Iterations	1
Interaction Zone Radius (px)	3
Number of Monte Carlo Simulations	1000
Max Allowed Overlap Ratio	0.1
Nematic Exclusion Size (px)	50
Max Void Contact Ratio (Rim Rejection)	0.4

Table S6 Output parameters determined by batch processing of cross-section images of PLA/BC-*g*-PLA5 using the custom-written Python script GPU_Void-Particle_Analyzer.

Metric	Value / Mean	Std Dev
Total Particles Analyzed	22078.0000	N/A
Total Voids Analyzed	12383.0000	N/A
Total Nematic Particles Included	2990.0000	N/A
Total Nematic Voids Included	308.0000	N/A
Void Area Fraction (%)	1.0636	0.4088
Particle Area Fraction (%)	5.6057	0.9312
Mean % Voids Touching Particles (All)	74.9425	8.5134
Mean P-Value (Touching, All)	0.0000	0.0000
Mean Nematic Order (Voids)	0.7503	0.0577
Mean Nematic Order (Particles)	0.4122	0.0617
Mean Delta Theta (deg)	3.3887	2.9037
Mean Nematic Coupling (S_coupled)	0.9886	0.0158
Mean Z-Score (All Voids)	-16.6513	3.2167
Global Z-Score (All Voids, Stouffer)	-47.0968	N/A
Global P-Value (All Voids, Stouffer)	0.0000	N/A
Mean % Voids Touching Particles (Large >50px)	92.3850	7.3468
Mean P-Value (Touching, Large)	0.0066	0.0079
Mean Z-Score (Large Voids >50px)	-1.9375	1.0434
Global Z-Score (Large Voids >50px, Stouffer)	-5.4801	N/A
Global P-Value (Large Voids >50px, Stouffer)	0.0000	N/A
Area px (void)	10.7342	18.3583
Area um^2 (void)	0.0035	0.0059
Eq. Diameter um (void)	0.0574	0.0335
Feret Diameter um (void)	0.0705	0.0726
Circularity (void)	2.4588	1.9781
Convexity (void)	0.9759	0.0417
Solidity (void)	2.2110	1.4457
Area px (particle)	31.7300	34.1283
Area um^2 (particle)	0.0103	0.0110
Eq. Diameter um (particle)	0.1063	0.0420
Feret Diameter um (particle)	0.1337	0.0935
Circularity (particle)	1.1040	0.3511
Convexity (particle)	0.9639	0.0621
Solidity (particle)	1.5176	0.3543

Table S7 Parameters used for the fitting of the PLA/BC-*g*-PLA10 cross-section images using the custom-written Python script GPU_Void-Particle_Analyzer.

Parameter	Value
Thresholding Method	Matrix-Capped Difference (Voids) + Adaptive (Particles)
Min Void Area (px)	2
Min Particle Area (px)	11
Large Void Threshold (px)	50
Adaptive Block Size (px)	111
Matrix Cap Multiplier	1.1
Void Depth Threshold	1
Adaptive C Particles	5
Morphological Iterations	1
Interaction Zone Radius (px)	3
Number of Monte Carlo Simulations	1000
Max Allowed Overlap Ratio	0.1
Nematic Exclusion Size (px)	50
Max Void Contact Ratio (Rim Rejection)	0.4

Table S8 Output parameters determined by batch processing of cross-section images of PLA/BC-*g*-PLA10 using the custom-written Python script GPU_Void-Particle_Analyzer.

Metric	Value / Mean	Std Dev
Total Particles Analyzed	235306.0000	N/A
Total Voids Analyzed	76211.0000	N/A
Total Nematic Particles Included	14816.0000	N/A
Total Nematic Voids Included	1174.0000	N/A
Void Area Fraction (%)	1.4285	0.2299
Particle Area Fraction (%)	10.6491	0.8050
Mean % Voids Touching Particles (All)	70.8414	3.6045
Mean P-Value (Touching, All)	0.0028	0.0166
Mean Nematic Order (Voids)	0.3064	0.1285
Mean Nematic Order (Particles)	0.2576	0.0793
Mean Delta Theta (deg)	17.8623	16.9713
Mean Nematic Coupling (S_coupled)	0.7132	0.4384
Mean Z-Score (All Voids)	-1.4769	2.3344
Global Z-Score (All Voids, Stouffer)	-8.7372	N/A
Global P-Value (All Voids, Stouffer)	0.0000	N/A
Mean % Voids Touching Particles (Large >50px)	90.8131	8.7210
Mean P-Value (Touching, Large)	0.5960	0.3018
Mean Z-Score (Large Voids >50px)	2.3483	1.1522
Global Z-Score (Large Voids >50px, Stouffer)	13.8926	N/A
Global P-Value (Large Voids >50px, Stouffer)	1.0000	N/A
Area px (void)	10.2478	12.6176
Area um^2 (void)	0.0033	0.0041
Eq. Diameter um (void)	0.0584	0.0284
Feret Diameter um (void)	0.0628	0.0489
Circularity (void)	2.3340	1.7426
Convexity (void)	0.9811	0.0337
Solidity (void)	2.3370	1.4172
Area px (particle)	24.7434	16.2281
Area um^2 (particle)	0.0080	0.0052
Eq. Diameter um (particle)	0.0974	0.0264
Feret Diameter um (particle)	0.1139	0.0544
Circularity (particle)	1.1647	0.2989
Convexity (particle)	0.9756	0.0432
Solidity (particle)	1.5747	0.3062

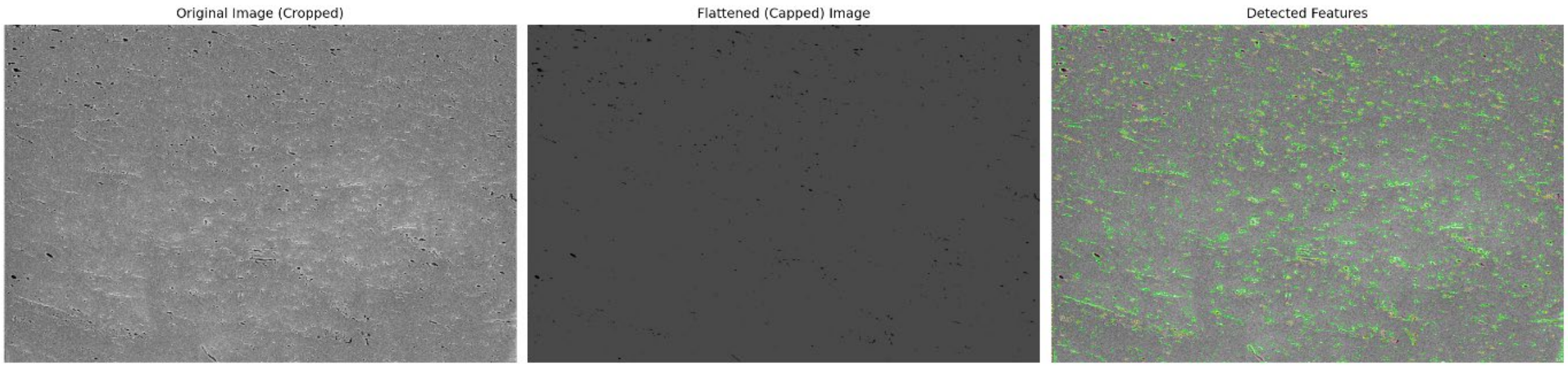

Fig. S5 Example overlay and original images from the image analysis of PLA/BC-*g*-PLA5.

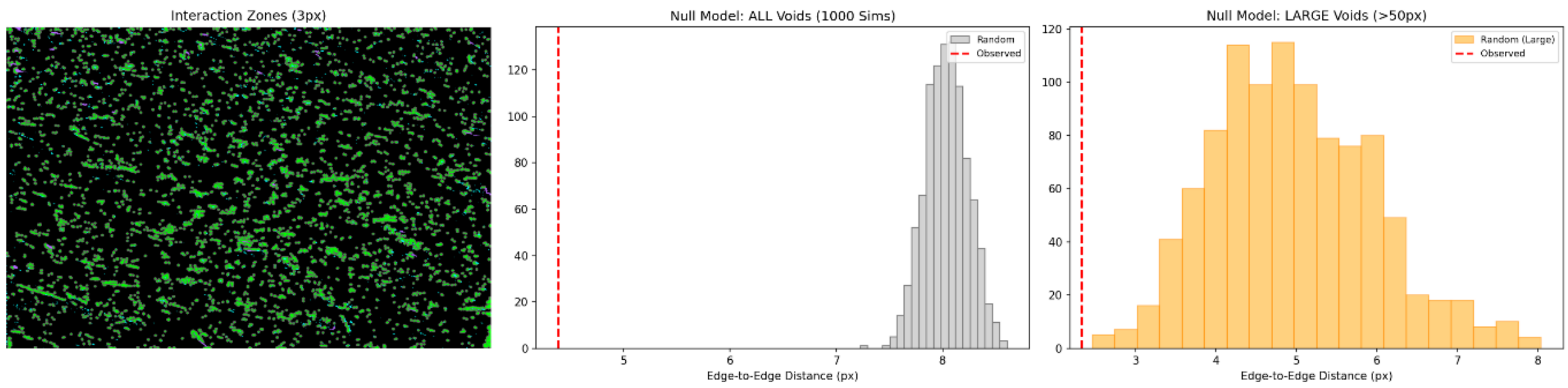

Fig S6 Example overlay of detected interaction zones and statistical simulation of void-particle proximity of PLA/BC-*g*-PLA5.

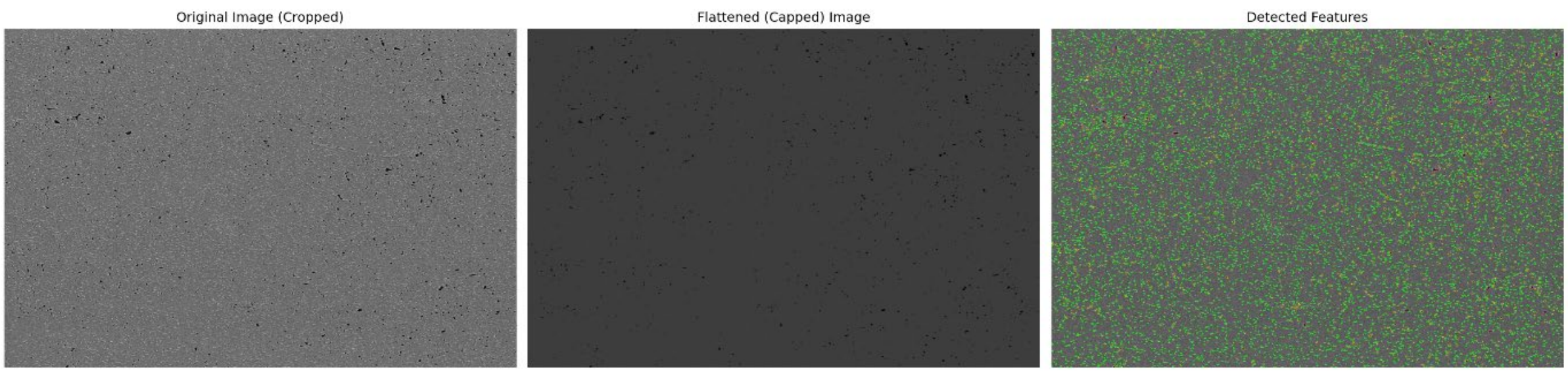

Fig. S7 Example overlay and original images from the image analysis of PLA/BC-*g*-PLA10.

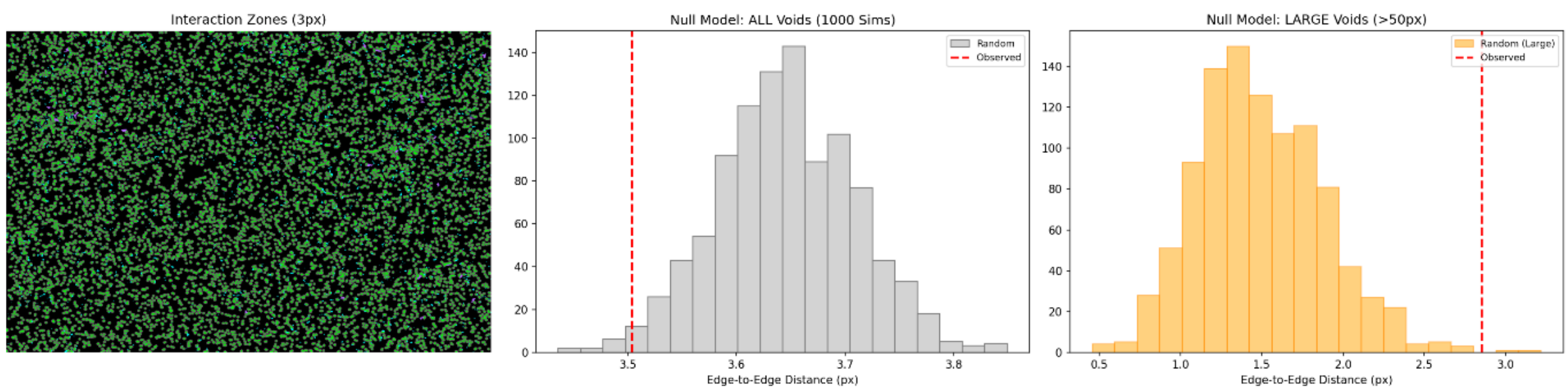

Fig. S8 Example overlay of detected interaction zones and statistical simulation of void-particle proximity of PLA/BC-*g*-PLA10.
